# Trajectories of family and employment stress associated with cerebrovascular accidents

**DOI:** 10.11606/s1518-8787.20210550033253

**Published:** 2021-11-23

**Authors:** María Pilar Jerez, Ignacio Madero-Cabib

**Affiliations:** I Pontificia Universidad Católica de Chile Magíster en Salud Pública Santiago Chile Magíster en Salud Pública. Pontificia Universidad Católica de Chile. Santiago, Chile; II Pontificia Universidad Católica de Chile Instituto de Sociología Departamento de Salud Pública Santiago Chile Pontificia Universidad Católica de Chile. Instituto de Sociología & Departamento de Salud Pública. Santiago, Chile; III Millennium Nucleus for the Study of the Life Course and Vulnerability Santiago Chile Millennium Nucleus for the Study of the Life Course and Vulnerability (MLIV). Santiago, Chile

**Keywords:** Stroke, Risk Factors, Financial Stress, Burnout, Professional, Socioeconomic Factors

## Abstract

**OBJECTIVES:**

Reconstruct types of simultaneous stress trajectories in the family and employment domain at different stages of life and estimate their association with cerebrovascular accident (CVA) in old age.

**METHODS:**

We used a retrospective, face-to-face, representative survey of people aged 65 to 75 years in the city of Santiago, Chile, (n = 802). We performed a multichannel sequence analysis to reconstruct family and employment stress trajectory types at various life stages and then used logistic regression models to estimate the association of these trajectory types with CVA in old age, controlled for traditional cardiovascular risk factors.

**RESULTS:**

Four representative types of family and employment stress trajectories were identified: (1) Absence of family and employment stress, (2) Absence of family stress, persistent employment stress, (3) Absence of family stress, out of the labor market, and (4) Persistent family stress, absence of employment stress. The 61.7% of the sample followed trajectories marked by the permanent presence of family and/or employment stress. Likewise, 18.3% had a trajectory characterized by prolonged absence from the labor market. Individuals with persistent family or employment stress trajectories, as well as those with extended periods of inactivity, are more at risk of developing CVA.

**CONCLUSIONS:**

Stress is a risk factor for cardiovascular disease experienced by many people at different stages and domains of life on a prolonged basis. Consequently, prevention systems for this type of chronic diseases should emphasize the highly harmful effects of daily and cumulatively stressful life experiences. This could mitigate the multiple health and financial consequences associated with CVA.

## INTRODUCTION

Cardiovascular diseases are a group of disorders of the circulatory and coronary systems that occur regularly in adulthood and old age^1^ and are currently the leading cause of death worldwide, accounting for 17.8 million deaths^2^. Among cardiovascular diseases, cerebrovascular accident (CVA) is the second leading cause of death in men and women (11.9 million deaths)^2^ and the third leading cause of disability in adults^3^.

Approximately 90% of CVA can be attributed to modifiable risk factors^3^. Epidemiological evidence indicates that these factors include smoking, high blood pressure, high cholesterol levels, diabetes mellitus, obesity, physical inactivity, alcohol consumption and inadequate dietary patterns^4^. However, there is also an emphasis on the growing influence of psychosocial, environmental and behavioral factors. Among these, stress is a crucial determinant^5^, as it is associated with the development of premorbid processes that lead to the occurrence of CVA^6^. Specifically, acute and long-term stressful experiences can trigger pathological cardiac events and are associated with an increased risk of developing CVA and greater associated mortality^6–9^.

From life course epidemiology, the development of CVA is a result of multiple exposures to adverse social, physical and psychological conditions during different stages of people's lives^10–12^. For example, the accumulation of adverse experiences during childhood, such as parental illness, prolonged family conflict or financial problems showed to have a significantly positive effect on the risk of CVA later in life^11^. On the other hand, some studies suggest that facing stressful events during adulthood, whether earthquakes^13^ or family and interpersonal events such as the loss of a partner, as well as isolation of community, are associated with an increase in CVA^8,14^.

In addition to stressful family experiences, another domain in which people often face stressful situations is the labor market. Evidence suggests that those exposed to multiple employment stressors, such as a high workload and long working hours, are at increased risk of developing CVA, especially when stress exposures are frequent over time^15^.

Despite the advances in relation to the effect of family and employment stressors on the risk of CVA, there are at least three aspects that still require further elaboration in this study field. First, most research so far considers measurements of stress and CVA at one or two life stages. This precludes understanding family or employment stress patterns along people's lives, as well as their cumulative effect on CVA.

Second, despite the growing evidence on the consequences of family and employment stressors on CVA, most studies focused on only one domain separately to measure the presence of stress. Consequently, the studies have been ignored the simultaneous impact of experiencing family and employment stress on this type of pathology. The simultaneous effect of family and employment stress is relevant because the harmful effect of the presence of stress in one of these domains could be compensated by the absence of stress in the other; or the effect on CVA could be greater due to the presence of stress in both life domains.

Third, the growing research in this field almost exclusively occurs in high-income countries with better health promotion and disease prevention-oriented health systems, such as Finland and the United Kingdom^8,15^. After an exhaustive review of the current evidence, we did not find any studies on trajectories of employment or family stress and CVA in developing or middle-income countries, such as those in the Latin American. Understanding the relationship between stress trajectories and CVA could allow us to reorient public policies for the prevention of this type of disease in Latin American countries as well, directing efforts towards stress mitigation in different domains and stages of people's lives.

Considering these knowledge gaps, the present study has two main objectives: reconstruct different types of simultaneous stress trajectories in the family and employment domains during the life of individuals in Chile; and estimate the association of these types of trajectories on the presence of CVA in old age.

## METHODS

### Data and Sample

This study used data from the survey “*Curso de vida y vulnerabilidad en personas mayores en Santiago, Chile*” (Life course and vulnerability in older people in Santiago, Chile), which collected retrospective information on multiple life domains, such as residential, educational and occupational histories, health risk habits, marital and fertility patterns, financial vulnerabilities, and health status in old age. The survey was face-to-face, conducted between March and August 2019, obtaining representative information from 802 people aged 65 to 75 years old residing in Santiago, Chile.

We conducted the data collection following the quality standards defined by the American Association for Public Opinion Research^16^. The selection of the sample of individuals was random, with a maximum variance (p = 0.5), a confidence level of 95%, and an estimated error of ± 3.5 points for an infinite population. To mitigate possible non-response bias in the sample selection, we adjusted the database by a weighting factor that corrects our estimates for known population characteristics of this age group in Santiago, such as areas of residence, educational level and sex.

**Figure 1:**
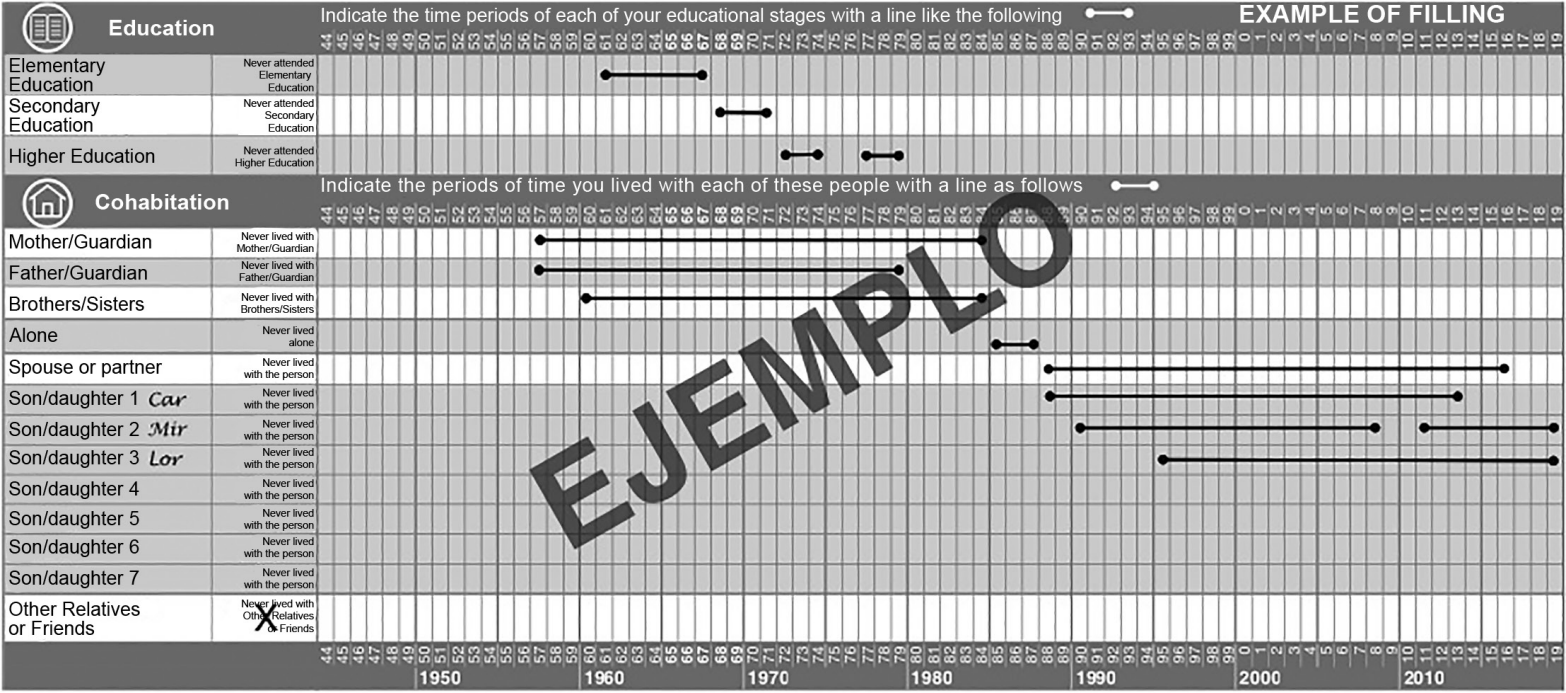
Life course calendar used in this study.

A retrospective instrument called “life course calendar”^17,18^ was used to collect the data, specially designed to help the interviewees remember and chronologically organize the episodes of their lives with approximate dates of occurrence. Figure 1 shows an example of the life course calendar used in this research. The time unit used in this tool corresponded to annual information. Given that a major limitation of retrospective measurement instruments corresponds to memory bias, the life course calendar used in this study was created by addressing three key aspects of autobiographical memory functioning. First, it includes a timeline that forces people to think retrospectively about their lives chronologically. Second, it requires individuals to first recall experiences in key life domains (for example, births, marriages, divorces, widowhood) and then relate them to other experiences in the remaining domains, thereby increasing the accuracy of the reconstruction of the past. And third, respondents have the opportunity to add information to the life course calendar, even if it means moving backwards or forwards in the timeline^17,18^.

### Variables

#### Independent Variable: Family and employment stress trajectories

To reconstruct types of family and employment stress trajectories, we considered life course calendar questions in which we asked to each participant to identify periods in their lives when they experienced family and employment stress according to their own criteria. Specifically, while for the family domain we asked each participant to mark the years in which they “*felt stress due to family causes*”; in the employment domain we asked them to answer “yes” or “no” to the question “*Was it generally a stressful employment?*” for each of the jobs they had during their life. From these questions, we first created the variable “employment stress”, which measured the categories “presence of stress”, “absence of stress” or “out of the labor market” in the case of a year in which the person was outside the labor force. Secondly, we created the variable “family stress”, which similarly identified for each year of people's lives the categories: “presence of stress” and “absence of stress”.

#### Dependent Variable: Cerebrovascular accident

The dependent variable in our study corresponds to having experienced a cerebrovascular accident or not. Following international standards, the participants were asked: “*Has a doctor ever told you that you have or had any of the health problems listed on this card? Remember that they may be health problems that you currently have or had in the past.*” Among the options was “Stroke or cerebral vascular disease”.

#### Control Variables: Sociodemographic and traditional CVA risk factors

Considering studies conducted in high-income countries, the analyses were first adjusted for the following four sociodemographic variables: age, sex (woman, man), educational level (elementary, secondary or higher education), and health coverage (public, private). The latter variable is a proxy indicator of the socioeconomic level of the respondent, since people with a better financial situation have access to private health coverage. Our analyses were also adjusted for traditional CVA risk factors, such as body mass index (obese, non-obese); physical activity (one or more times per week / once a month or less); frequency of consumption of (1) dairy, (2) legumes, beans or eggs and (3) meat, fish or poultry (one or more times per week / less than once a week); current smoking (presence/absence); and diagnosis of hypertension (presence/absence).

### Statistical Methods

First, we employed multichannel sequence analysis^19–21^ to reconstruct representative types of simultaneous trajectories of family and employment stress during the life course. Multichannel sequence analysis allows comparing similarities and differences between life trajectories in two or more life domains (in this case employment stress and family stress), by estimating the *distance* between these trajectories. Distance is the cost of transforming the trajectory of an individual in a specific domain to the trajectory of another individual in that domain, through two specific operations: status substitution from which trajectories are measured, and insertion or elimination of this status^22^.

Once the distances between all individual trajectories are obtained, they are inserted into a distance matrix, on which a hierarchical cluster analysis is performed using Ward's method^23^, which allowed us to group trajectories similar to each other (with less distance between them) into different clusters or representative types of family and employment stress trajectories. Finally, to determine the most robust number of trajectory types representative of the diversity of possible trajectories in the sample, we used four statistical selection criteria: *average silhouette width* (ASW), *point biserial correlation* (PBS)*, Hubert's gamma* (HG) and *Hubert's C* (HC)^24^. The scores of these criteria ranges from −1 to 1, or from 0 to 1. To ensure comparability between the criteria, their scores were standardized. In the case of ASW, PBC and HG, higher values mean a better solution, while lower values in HC indicate a better solution.

After identifying the most robust number of family and employment stress trajectory types, this became the main independent variable in a logistic regression model in which we measure its association with CVA, adjusting for the control variables indicated previously. We conducted the analyses in this study in the statistical software R^25^, specifically with the statistical package TraMineR^22^ to estimate multichannel sequence analyses, and the statistical package survey^26^ to work with a weighted database.

## RESULTS

### Sample Characteristics

The estimated weighted univariate proportions of the dependent and control variables are in Table 1. Among other results, we observed that 6.4% of the sample reported having experienced a CVA, women were 56.7% of those analyzed, 30.3% reported obesity, and 66% had a diagnosis of hypertension.

**Table 1:** Weighted univariate proportions of dependent and control variables.

		%
CVA Diagnosis		
	Absence	93.6
	Presence	6.4
Sex		
	Man	43.3
	Woman	56.7
Educational level		
	Secondary or higher	61.5
	Elementary	38.5
Health coverage		
	Public	88.0
	Private	11.2
Dairy consumption		
	Less than once a week	12.3
	One or more times per week	87.2
Consumption of meat, fish or poultry		
	Less than once a week	1.7
	One or more times per week	98.3
Consumption of legumes, beans or eggs		
	Less than once a week	3.7
	One or more times per week	96.2
Physical activity		
	Once a month or less	56.6
	One or more times per week	43.4
Smoking		
	Absence	81.1
	Presence	17.3
Hypertension		
	Absence	66.0
	Presence	34.0
Body mass index		
	Not obese	69.7
	Obese	30.3

CVA: cerebrovascular accident. Note: The only variable that presented missing values was “Smoking” with 1.6%.

### Family and Employment Stress Trajectories

**Figure 2:**
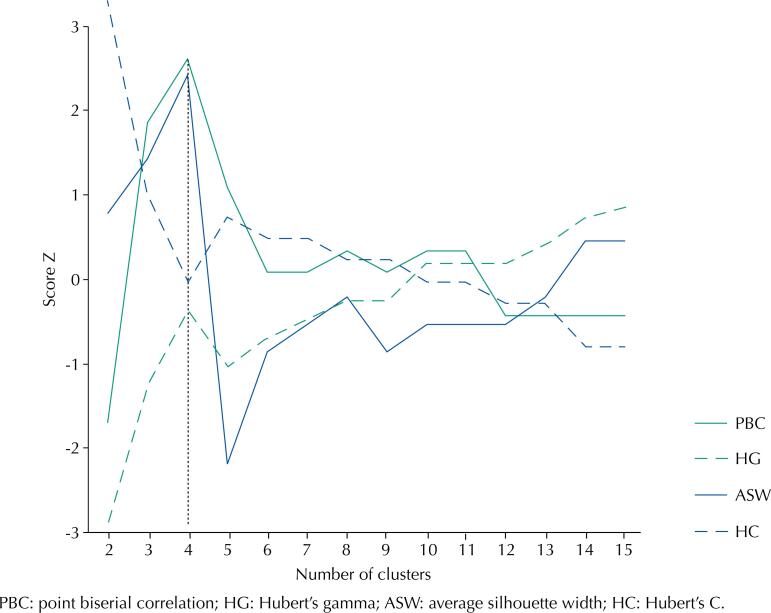
Cluster selection criteria.

Note: Higher values for PBC, HG and ASW, and lower values for HC, group a better and more robust solution for the number of clusters.

Figure 2 suggests that, based on the aforementioned selection criteria, four clusters correspond to the most robust number of trajectory types to adequately represent the diversity of family and employment stress patterns in the sample studied.

**Figure 3:**
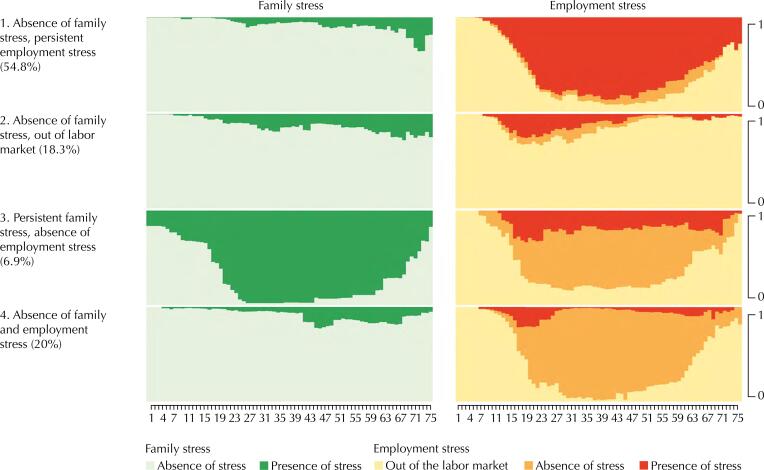
Chronogram graphs of the four types of family and employment stress trajectories.

Note: The weighted proportion of each of the family and employment stress trajectory types is in parentheses.

**Figure 4:**
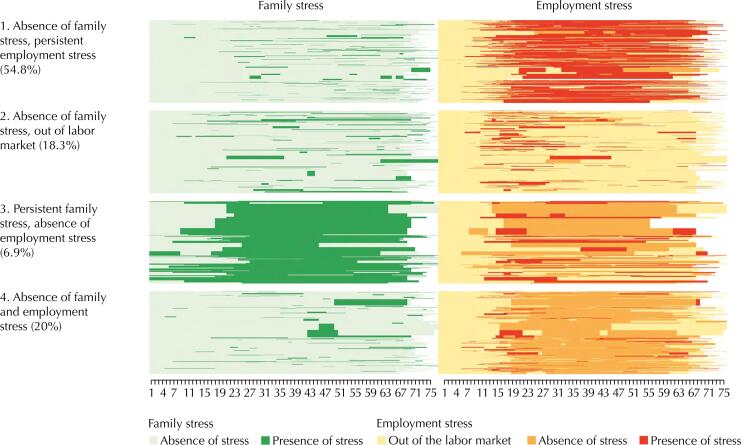
Graphs of individual sequences of the four types of family and employment stress trajectories.

Note: The weighted proportion of each of the family and employment stress trajectory types is parentheses.

Figures 3 and 4 detail the four types of trajectories in two types of graphs: chronogram graphs and individual sequence graphs, respectively. In each of these graphs, the trajectory of family stress and employment stress is in different columns. On the left of the graphs are represented the names and weighted proportions of each type of trajectory, and on the right the family and employment stress status used to reconstruct these trajectories. In the chronogram graph, the X-axis represents the age of individuals; and the Y-axis, the proportion (0 to 1) of individuals in different types of stress status over time. In the graph of individual sequences, the X-axis also indicates age, but the Y-axis shows the specific trajectory of each individual classified in a trajectory type (the number of lines is equal to the number of persons classified in the type).

The first type, “Absence of family stress, persistent employment stress”, includes 54.8% of the sample and corresponds to people who mostly described not having felt stress due to family causes, but permanently due to employment causes. This group reports having experienced employment stress mostly from the age of 20 years old constantly, which decreases after the age of 50 years when the leaving from the labor market begins to increase progressively.

The second type of trajectory, “Absence of family stress, out of the labor market”, gathers 18.3% of the sample, characterized by people who most of the time in their lives were without a paid work, and who did not report having felt stress due to family causes. In this group, some people report having suffered employment stress in early adulthood (from 18 to 30 years of age), but then left the labor market.

The third type of trajectory, “Persistent family stress, absence of employment stress”, included 6.9% of the sample and was composed of individuals who mostly reported no employment stress, but reported family stress. In this type, even some people are classified as having felt family stress since early childhood, increasing markedly after the age of 18 and remaining constant until after 60 years old.

Finally, the fourth type of trajectory “Absence of family and employment stress” includes 20% of the sample and consists of people who mostly described not having felt stress due to family or employment stress in their lives.

### Trajectories of Family and Employment Stress and CVA

Table 2 shows the result of three logistic regression models: the first includes only the types of stress trajectories, the second adds sociodemographic variables, while the third also considers traditional CVA risk factors. The results are reported in odds ratios (OR) with their respective confidence intervals and levels of statistical significance.

**Table 2:** Logistic regression on cerebrovascular accident.

		Odds ratios	95% confidence intervals	*Odds ratios*	95% confidence intervals	Odds ratios	95% confidence intervals
Type of employment and family stress trajectories							
	Absence of family and employment stress						
	Absence of family stress, persistent employment stress	2.42	0.77—7.56	3.16^d^	0.89–11.17	3.38^d^	1.00–11.50
	Absence of family stress, out of the labor market	9.18^b^	2.16—39.00	8.54^b^	1.80–40.50	8.28^b^	1.86–36.93
	Persistent family stress, absence of employment stress	12.64^a^	3.53—45.23	12.90^a^	2.96–56.14	11.87^b^	2.80–50.38
Sociodemographic variables							
Age				1.09	0.87–1.36	1.11	0.90–1.37
Sex							
	Man	–		–		–	
	Woman			0.94	0.44–2.01	0.80	0.37–1.83
Educational level							
	Secondary or higher	–		–		–	
	Elementary			1.95^d^	0.93–4.11	1.50	0.79–2.84
Health coverage							
	Public	–		–		–	
	Private			0.00^a^	0.00–0.00	0.00^a^	0.00–0.00
Traditional CVA risk factors							
Body mass index							
	Not obese	–		–		–	
	Obese					1.72	0.74–4.00
Physical activity							
	Once a month or less	–		–		–	
	One or more times per week					0.90	0.25–3.19
Dairy consumption							
	Less than once a week	–		–		–	
	One or more times per week					0.51	0.17–1.54
Consumption of legumes, beans or eggs							
	Less than once a week	–		–		–	
	One or more times per week					0.28^c^	0.09–0.86
Consumption of meat, fish or poultry							
	Less than once a week	–		–		–	
	One or more times per week					0.76	0.09–6.40
Smoking							
	Absence	–		–		–	
	Presence					1.21	0.55–2.67
Hypertension							
	Absence	–		–		–	
	Presence					1.74	0.68–4.46
Intercept		0.02		0.00		0.00	

a p < 0.001.

b p < 0.01.

c p < 0.05.

d p < 0.1. Note: Statistically significant effects marked in gray.

The model that considers adjustments for all control variables shows that the possibility of suffering a CVA is significantly higher in all types of stress trajectories, compared to those subjects grouped in the “Absence of family and employment stress” trajectory. Specifically, in those subjects that compose the trajectory “Absence of family stress, persistent employment stress”, the probability of suffering a CVA is more than twice higher (OR = 3.38; p < 0.1). While for those classified in the “Absence of family stress, out of the labor market” trajectory, the probability is more than seven times higher (OR = 8.28; p < 0.01), and for those who followed the trajectory “Persistent family stress, absence of employment stress” the probability is more than ten times higher (OR = 11.87; p < 0.01). In relation to the control factors, having private health coverage, as well as the consumption of legumes, beans or eggs more than once a week, reduces the risk of CVA.

Additionally, based on previous evidence on differences between men and women regarding cerebrovascular disease^27^, we conducted a sensitivity analysis including interaction effects between trajectory types and sex on CVA risk (see Table 3). As can be seen, the model that considers all controls suggests that only in the relationship between the trajectory “Persistent family stress, absence of employment stress” and CVA, men show a significantly higher effect than women (OR = 8.69; p < 0.5).

**Table 3:** Logistic regression on cerebrovascular accident (interaction effects between trajectory types and sex).

		Odds ratios	95% confidence intervals	Odds ratios	95% confidence intervals	Odds ratios	95% confidence intervals
Type of employment and family stress trajectories							
	Absence of family and employment stress	–		–		–	
	Absence of family stress, persistent employment stress	1.31	0.23–7.35	1.94	0.28–13.18	2.27	0.33–15.58
	Absence of family stress, out of the labor market	7.50	0.59–95.57	7.71	0.47–125.29	8.98	0.59–95.57
	Persistent family stress, absence of employment stress	8.32^b^	1.52–45.64	9.15^b^	1.37–61.18	8.69^b^	1.22–61.83
Type of employment and family stress trajectories * Sex							
	Absence of family stress, persistent employment stress * Woman	3.54	0.30–42.16	2.72	0.20–36.25	2.28	0.17–28.86
	Absence of family stress, out of labor market * Woman	2.27	0.09–46.06	1.64	0.06–44.34	1.27	0.04–36.28
	Persistent family stress, absence of employment stress * Woman	2.06	0.15–33.96	2.00	0.14–28.25	1.92	0.12–29.51
Sociodemographic variables							
Age				1.09	0.88–1.36	1.12	0.92–1.37
Educational level							
	Secondary or higher	–		–		–	
	Elementary			1.87	0.85–4.01	1.44	0.74–2.80
Health coverage							
	Public	–		–		–	
	Private			0.00^a^	0.00–0.00	0.00^a^	0.00–0.00
Traditional CVA risk factors							
Body mass index							
	Not obese	–		–		–	
	Obese					1.70	0.75–3.88
Physical activity							
	Once a month or less	–		–		–	
	One or more times per week					0.91	0.26–3.25
Dairy consumption							
	Less than once a week	–		–		–	
	One or more times per week					0.51	0.17–1.51
Consumption of legumes, beans or eggs							
	Less than once a week	–		–		–	
	One or more times per week					0.29^b^	0.09–0.88
Consumption of meat, fish or poultry							
	Less than once a week	–		–		–	
	One or more times per week					0.76	0.09–6.52
Smoking							
	Absence	–		–		–	
	Presence					1.25	0.58–2.69
Hypertension							
	Absence	–		–		–	
	Presence					1.73	0.68–4.40
Intercept		0.02		0.00		0.00	

a p < 0.001.

b p < 0.05. Note: Statistically significant effects marked in gray.

## DISCUSSION

Using a retrospective survey, representative of people aged 65 to 75 years in the city of Santiago, Chile, and based on a life course approach, this study analyzed the association between different types of simultaneous family and employment stress trajectories and the presence of CVA in old age. The results of this study indicate that a significant proportion of older people in Chile (61.7%) followed trajectories marked by the permanent presence of family and/or employment stress. Likewise, a not smaller proportion (18.3%) had a trajectory characterized by prolonged absence from the labor market.

In terms of the association with CVA, we observed that trajectories characterized by permanent exposure to stressful family and/or employment situations during life are significantly associated with the presence of this cardiovascular disease. These findings are consistent with previous cross-sectional studies. For example, in a meta-analysis of 14 studies involving 10,130 patients diagnosed with CVA, a 33% increased risk of this disease was estimated among those who reported experiencing family, employment, or financial stress^28^.

Our results show that the type of trajectory characterized by the persistent presence of family stress, but with the absence of employment stress, is more associated with the presence of CVA (indicating a risk more than 10 times higher than the type of trajectory “Absence of family and employment stress”). This result is in line with previous studies that have observed a relevant effect on the presence of CVA in those individuals facing stressful marital and interpersonal relationships^29^. In addition, our sensitivity analysis (Table 3) show that this type of trajectory is significantly riskier in men than in women. In this regard, some researchers have pointed to an increased risk of CVA in groups that had experienced stress associated with divorce or death of a partner, particularly in men^14^.

On the other hand, our study evidences a strong association with CVA among people who report not having suffered stress due to family causes, but were out of the labor market for prolonged periods of time. These results are also consistent with previous cross-sectional studies, which show that those who stop working for pay have more than twice the risk of CVA (as well as other cardiovascular diseases such as acute myocardial infarction), especially if the inactivity occurs involuntarily^27,30,31^. In addition, other studies in Latin America indicate a higher mortality associated with CVA in inactive subjects.^32^. One possible explanation for this association is that, especially in countries such as Chile, having a continuous work implies obtaining a financial income that allows satisfying individual needs such as access to better preventive health services and pharmacological treatments^33–35^.

### Limitations

This study has some limitations that should be considered when interpreting findings. First, due to inevitable memory bias, respondents may have inaccurately reported early life experiences. To mitigate this bias, the life course calendar employed in this study incorporated methodological innovations aimed at strengthening autobiographical memory, such as the decomposition of life domains into different subcategories, the use of illustrative visual supports, and the orientation of a properly trained interviewer in the process of filling out the questionnaire. Second, in relation to the sample, it is relevant to consider that it is representative of the capital, but not of all of Chile. This is important, since the centralized nature of the country means that access to health services is greater and of better quality in the capital than in the rest of the regions. A final relevant limitation refers to the fact that the report of stress by the participants depends closely on their perception of the concept of “stress”. We know, however, that there is no single definition of this term, as it encompasses psychological, emotional, motivational and cognitive aspects. Likewise, when identifying employment stress, participants may have considered different aspects than when reporting family stress.

## IMPLICATIONS AND CONCLUSION

This study has relevant implications for the field of cardiovascular diseases. CVA are highly prevalent pathologies, with catastrophic health consequences and highly associated with modifiable risk factors, including stress. In order to reduce mortality and the consequences that these pathologies have on the population, it is essential to consider these risk factors and strengthen health systems with a promotional and preventive approach, focusing on reducing health inequities, and not only on optimizing medical technologies for treatment and rehabilitation.

As this study suggests, stress is a risk factor experienced on a prolonged basis by a high proportion of people at different stages and domains of life. Consequently, it is necessary to adopt a promotional and preventive approach that emphasizes the adverse effects of experiencing daily and cumulatively stressful situations. The formulation of public policies aimed at reducing stress could contribute to mitigating the risk of CVA in traditionally susceptible populations.
