# Supplementary material for: Trajectories of family and employment stress associated with cerebrovascular accidents
Source: Rev Saude Publica. 2021 Nov 23;55:101. doi: 10.11606/s1518-8787.20210550033253 (PMC8621564; doi:10.11606/s1518-8787.20210550033253)
Supplement: Translation

# Translation: Trayectorias de estrés familiar y laboral y su asociación con accidentes cerebrovasculares

**Authors:** María Pilar Jerez, Ignacio Madero-Cabib

**Affiliations:** I Santiago Chile Magíster en Salud Pública. Pontificia Universidad Católica de Chile. Santiago, Chile; II Santiago Chile Pontificia Universidad Católica de Chile. Instituto de Sociología & Departamento de Salud Pública. Santiago, Chile; III Santiago Chile Millennium Nucleus for the Study of the Life Course and Vulnerability (MLIV). Santiago, Chile

**Keywords:** Accidente Cerebrovascular, Factores de Riesgo, Estrés Financiero, Agotamiento Profesional, Factores Socioeconómicos

## Abstract

**OBJETIVOS:**

Reconstruir tipos de trayectorias de estrés simultáneo en el dominio familiar y laboral en diferentes etapas de la vida y estimar su asociación con accidentes cerebrovasculares (ACV) en la vejez.

**MÉTODOS:**

Se utilizó una encuesta retrospectiva, cara a cara, y representativa de personas de 65 a 75 años en la ciudad de Santiago de Chile (n = 802). Se empleó un análisis de secuencias multicanal para reconstruir tipos de trayectorias de estrés familiar y laboral en diversas etapas de la vida y luego se utilizaron modelos de regresión logística para estimar la asociación de estos tipos de trayectoria con ACV en la vejez, controlado por factores tradicionales de riesgo cardiovascular.

**RESULTADOS:**

Se identificaron cuatro tipos representativos de trayectorias de estrés familiar y laboral: (1) Ausencia de estrés familiar y laboral, (2) Ausencia de estrés familiar, estrés laboral persistente, (3) Ausencia de estrés familiar, fuera de mercado laboral, y (4) Estrés familiar persistente, ausencia de estrés laboral. El 61,7% de la muestra siguió trayectorias marcadas por la presencia permanente de estrés familiar y/o laboral. Asimismo, el 18,3% tuvo una trayectoria caracterizada por la ausencia prolongada del mercado del trabajo. Las personas con trayectorias de estrés familiar o laboral persistente, así como aquellas con períodos extensos de inactividad, tienen más riesgo de desarrollar ACV.

**CONCLUSIONES:**

El estrés es un factor de riesgo de enfermedades cardiovasculares que experimenta de manera prolongada muchas personas en distintas etapas y dominios de la vida. En consecuencia, los sistemas de prevención de este tipo de enfermedades crónicas deben enfatizar los efectos altamente nocivos de enfrentar cotidiana y acumulativamente experiencias de vida estresantes. Esto a su vez podría mitigar las múltiples consecuencias sanitarias y financieras asociadas al ACV.

## INTRODUCCIÓN

Las enfermedades cardiovasculares son un grupo de desórdenes del sistema circulatorio y coronario que se manifiestan regularmente en la adultez y la vejez^1^, y representan actualmente la principal causa de muerte en el mundo, alcanzando 17,8 millones de muertes^2^. Entre las enfermedades cardiovasculares, el accidente cerebrovascular (ACV) es la segunda causa de muerte en hombres y mujeres (11,9 millones de muertes)^2^, y la tercera causa de discapacidad en adultos^3^.

Se estima que aproximadamente el 90% de los ACV pueden ser atribuidos a factores de riesgo modificables^3^. La evidencia epidemiológica ha destacado que entre estos factores se encuentran el hábito tabáquico, presión arterial elevada, niveles nocivos de colesterol, diabetes mellitus, obesidad, inactividad física, consumo de alcohol y patrones dietéticos inadecuados^4^. Sin embargo, también se ha enfatizado la creciente influencia de factores de tipo psicosocial, del entorno y del comportamiento. Entre ellos, el estrés se considera un determinante crucial^5^, pues se asocia al desarrollo de procesos premórbidos que conducen a la aparición de ACV^6^. Específicamente, las experiencias estresantes agudas y de larga duración pueden desencadenar eventos cardíacos patológicos y se asocian a un mayor riesgo de desarrollar ACV y a una mayor mortalidad asociada^6–9^.

Desde la epidemiología de curso de vida, el desarrollo de ACV se ha entendido como el resultado de múltiples exposiciones a condiciones sociales, físicas y psicológicas adversas durante distintas etapas de la vida de las personas^10–12^. Por ejemplo, la acumulación de experiencias adversas durante la niñez, tales como enfermedades de los padres, conflictos familiares prolongados o problemas financieros, han mostrado un efecto significativamente positivo sobre el riesgo de ACV en etapas posteriores de la vida^11^. Por otra parte, algunas investigaciones sugieren que enfrentar eventos estresantes durante la adultez, ya sea terremotos^13^ o eventos familiares e interpersonales como la pérdida de la pareja, así como el aislamiento comunitario, inciden en un aumento de ACV^8,14^.

Además de las experiencias familiares estresantes, se ha descrito que otro de los dominios en el que las personas suelen enfrentar situaciones de estrés es el mercado laboral. La evidencia sugiere que aquellas personas expuestas a múltiples estresores laborales, por ejemplo una alta carga laboral y jornadas extensas de trabajo, presentarían mayor riesgo de desarrollar ACV, especialmente cuando las exposiciones a estrés son frecuentes en el tiempo^15^.

A pesar de los avances en relación al efecto de estresores familiares y laborales sobre el riesgo de ACV, existen al menos tres aspectos que aún requieren mayor elaboración en este campo de estudio. En primer lugar, hasta el momento la mayoría de las investigaciones consideran mediciones del estrés y de ACV en una o dos etapas de la vida. Esto impide entender patrones de estrés de tipo familiar o laboral a lo largo de la vida de las personas, así como su efecto acumulativo sobre el ACV.

En segundo lugar, pese a la creciente evidencia sobre las consecuencias de estresores familiares y laborales sobre ACV, la mayoría de los estudios se han centrado en uno u otro dominio por separado para medir la presencia de estrés. En consecuencia, se ha ignorado el impacto simultáneo de experimentar estrés familiar y laboral sobre este tipo de patologías. El efecto simultáneo de estrés por causas familiares y laborales es relevante por cuanto el efecto nocivo de la presencia de estrés en uno de estos dominios podría ser compensado por la ausencia de estrés en el otro; o por el contrario, el efecto sobre el ACV podría verse aumentado por la interacción de la presencia de estrés en ambos dominios de vida.

En tercer lugar, la creciente investigación en este campo ha sido conducida casi únicamente en países de altos ingresos, los que cuentan con sistemas sanitarios mejores y orientados a la promoción de la salud y prevención de enfermedades, tales como Finlandia y Reino Unido^8,15^. Luego de revisar exhaustivamente la evidencia actual, no se encontraron estudios sobre trayectorias de estrés laboral o familiar y ACV en países en desarrollo o de ingresos medios, como aquellos de la región latinoamericana. Comprender la relación entre trayectorias de estrés y ACV podría permitir reorientar las políticas públicas de prevención de este tipo de enfermedades también en los países latinoamericanos, dirigiendo esfuerzos hacia la mitigación del estrés en diferentes dominios y etapas de la vida de las personas.

Considerando estas brechas de conocimiento, el presente estudio se plantea dos objetivos principales: Reconstruir diferentes tipos de trayectorias de estrés simultáneo en el dominio familiar y laboral durante la vida de las personas en Chile; y estimar la asociación de dichos tipos de trayectorias sobre la presencia de ACV en la vejez.

## MÉTODOS

### Base de Datos

Este estudio utilizó datos de la encuesta “Curso de vida y vulnerabilidad en personas mayores en Santiago, Chile”, la cual recolectó información retrospectiva en múltiples dominios de la vida, tales como historias residenciales, educacionales y ocupacionales, hábitos de riesgo para la salud, patrones maritales y de fertilidad, vulnerabilidades financieras, y estados de salud en la vejez. La encuesta fue cara a cara, aplicada entre marzo y agosto del año 2019, y obtuvo información representativa de 802 personas de 65 a 75 años de edad que residían en Santiago de Chile.

La recopilación de datos se realizó siguiendo los estándares de calidad definidos por la Asociación Americana de Investigación de Opinión Pública^16^. La selección de la muestra de individuos fue aleatoria, con una varianza máxima (p = 0,5), un nivel de confianza del 95%, y un error estimado de ± 3,5 puntos para una población infinita. Para mitigar los posibles sesgos de no respuesta en la selección de la muestra, la base de datos fue ajustada por un factor de ponderación que corrige nuestras estimaciones por características poblacionales conocidas de este tramo de edad en Santiago, tales como zonas de residencia, nivel educacional y género.

Para recopilar los datos se utilizó el instrumento retrospectivo llamado “calendario de curso de vida”^17,18^, diseñado especialmente para ayudar a los entrevistados a recordar y organizar cronológicamente los episodios a lo largo de sus vidas con fechas aproximadas de ocurrencia. En la Figura 1 presentamos un ejemplo del calendario de curso de vida utilizado en esta investigación. La unidad de tiempo empleada en esta herramienta correspondió a información anual. Dado que una limitante importante de los instrumentos de medición retrospectivos corresponde al sesgo de memoria, el calendario de curso de vida utilizado en este estudio fue creado abordando tres aspectos claves del funcionamiento de la memoria autobiográfica. Primero, incluye una línea de tiempo que fuerza a las personas a pensar retrospectivamente sobre sus vidas de forma cronológica. Segundo, exige a los individuos que recuerden en primer lugar experiencias en dominios clave para la vida (por ejemplo, nacimientos, matrimonios, divorcios, viudez) para luego relacionarlos con otras experiencias en el resto de los dominios, aumentando así la precisión de la reconstrucción del pasado. Y tercero, entrega a los encuestados la oportunidad de agregar información al calendario, incluso si esto significa retroceder o avanzar en la línea de tiempo^17,18^.

**Figura 1:** Calendario de curso de vida utilizado en este estudio.

### Variables

#### Variable Independiente: Trayectorias de estrés familiar y laboral

Para reconstruir tipos de trayectorias de estrés familiar y laboral, se consideraron preguntas del calendario de curso de vida en las que los participantes debieron identificar períodos de sus vidas en que experimentaron estrés por motivos familiares y laborales de acuerdo a su propio criterio. Específicamente, mientras que para el dominio familiar a cada participante se le solicitó marcar los años en que “*sintió estrés por causas familiares”*, en el dominio laboral se le pidió que para cada uno de los trabajos en los que se había desempeñado durante su vida respondiera “sí” o “no” a la pregunta “*¿En general fue un trabajo estresante?”*. A partir de estas preguntas, se creó en primer lugar la variable “estrés laboral”, que midió para cada año de vida de las personas las categorías “presencia de estrés”, “ausencia de estrés” o “fuera del mercado laboral” en caso de ser un año en el que la persona no se encontrara empleada. En segundo lugar, se creó la variable “estrés familiar”, la cual de modo similar identificó para cada año de vida de las personas las categorías: “presencia de estrés” y “ausencia de estrés”.

#### Variable Dependiente: Accidente cerebrovascular

La variable dependiente de nuestro estudio corresponde a haber experimentado o no un accidente cerebrovascular. Siguiendo estándares internacionales, a los participantes se les consultó “*¿Alguna vez un médico le ha dicho que tiene o tenía alguno de los problemas de salud que figuran en esta tarjeta? Recuerde que pueden ser problemas de salud que tiene actualmente, o tuvo anteriormente*”*.* Entre las opciones se encontraba “*Derrame cerebral o enfermedad vascular cerebral*”.

#### Variables de Control: Factores sociodemográficos y de riesgo tradicional de ACV

Tomando en cuenta estudios realizados en países de altos ingresos, los análisis fueron ajustados primero por las siguientes cuatro variables sociodemográficas: edad, género (mujer, hombre), nivel educacional (primaria, secundaria o terciaria), y cobertura de salud (pública, privada). Esta última variable se considera un indicador *proxy* de nivel socioeconómico del entrevistado por cuanto las personas con mejor situación financiera acceden a cobertura privada de salud. Nuestros análisis se ajustaron también por factores de riesgo tradicionales de ACV, tales como índice de masa corporal (obeso, no obeso); actividad física (una o más veces por semana / una vez al mes o menos); frecuencia de consumo de (1) lácteos, (2) legumbres, alubias o huevos y (3) carne, pescado o ave (una o más veces por semana / menos de una vez por semana); hábito tabáquico actual (presencia/ausencia); y diagnóstico de hipertensión (presencia/ausencia).

### Métodos Estadísticos

En primer lugar, empleamos un análisis de secuencia multicanal^19–21^ para reconstruir tipos representativos de trayectorias simultáneas de estrés familiar y laboral a lo largo de la vida. El análisis de secuencia multicanal permite comparar similitudes y diferencias entre trayectorias de vida en dos o más dominios de la vida (en este caso estrés laboral y estrés familiar), por medio del cálculo de la *distancia* entre dichas trayectorias. La distancia se entiende como el costo de transformar la trayectoria de un individuo en un dominio específico a la trayectoria de otro individuo en dicho dominio, mediante dos operaciones concretas: substitución de estatus a partir de los que se miden las trayectorias, e inserción o eliminación de estos estatus^22^.

Cuando se obtienen las distancias entre las distintas trayectorias individuales, estas son introducidas en una matriz de distancias, sobre la cual se realiza un análisis de conglomerados jerárquico usando el método de Ward^23^, que nos permitió agrupar trayectorias similares entre sí (vale decir, con menor *distancia* entre ellas) en diferentes conglomerados o tipos representativos de trayectorias de estrés familiar y laboral. Finalmente, para determinar el número más robusto de tipos de trayectorias representativos de la diversidad de trayectorias posibles en la muestra, se utilizaron cuatro criterios estadísticos de selección: *average silhouette width* (ASW), *point biserial correlation* (PBS)*, Hubert's gamma* (HG) y *Hubert's C* (HC)^24^. Los puntajes de estos índices varían de −1 a 1, o de 0 a 1. Para asegurar la comparación entre los criterios, sus puntajes fueron estandarizados. En el caso de ASW, PBC y HG, valores mayores significan una mejor solución, mientras que valores menores en HC indican una mejor solución.

Una vez identificado el número de tipos más robusto de trayectoria de estrés familiar y laboral, este se convirtió en la principal variable independiente para medir la asociación con ACV en un modelo de regresión logística, el cual fue ajustado por las variables de control indicadas previamente. Los análisis de este estudio fueron conducidos en el *software* estadístico R^25^, específicamente con el paquete estadístico *TraMineR*^22^ para estimar análisis de secuencia multicanal, y el paquete estadístico *survey*^26^ para trabajar con base de datos ponderada.

## RESULTADOS

### Características de la Muestra

Las proporciones univariadas ponderadas de las variables dependientes y de control se muestran en la Tabla 1. Entre los datos más destacados observamos que el 6,4% de la muestra reportó haber experimentado ACV, las mujeres constituyeron el 56,7% de los analizados, un 30,3% reportó obesidad, y un 66% presencia de un diagnóstico de hipertensión.

**Tabla 1:** Proporciones univariadas ponderadas de variables dependientes y de control.

		%
Diagnóstico ACV		
	Ausencia	93,6
	Presencia	6,4
Género		
	Hombre	43,3
	Mujer	56,7
Nivel educacional		
	Secundaria o terciaria	61,5
	Primaria	38,5
Cobertura de salud		
	Pública	88,0
	Privada	11,2
Consumo de lácteos		
	Menos de una vez por semana	12,3
	Una o más veces por semana	87,2
Consumo de carne, pescado o ave		
	Menos de una vez por semana	1,7
	Una o más veces por semana	98,3
Consumo de legumbres, alubias o huevos		
	Menos de una vez por semana	3,7
	Una o más veces por semana	96,2
Actividad física		
	Una vez al mes o menos	56,6
	Una o más veces por semana	43,4
Hábito tabáquico		
	Ausencia	81,1
	Presencia	17,3
Hipertensión		
	Ausencia	66,0
	Presencia	34,0
Índice de masa corporal		
	No obeso	69,7
	Obeso	30,3

ACV: accidente cerebrovascular. Nota: La única variable que presentó valores perdidos fue “Hábito tabáquico” con un 1,6%.

### Trayectorias de Estrés Familiar y Laboral

La Figura 2 sugiere que, basados en los criterios de selección antes mencionados, cuatro conglomerados corresponden al número más robusto de tipos de trayectorias para representar adecuadamente la diversidad de patrones de estrés familiar y laboral en la muestra estudiada.

**Figura 2:** Criterios de selección de conglomerados.

Nota: Mayores valores para PBC, HG y ASW, y menores valores para HC, agrupan una mejor y más robusta solución para el número de conglomerados.

En las Figuras 3 y 4 se detallan los cuatro tipos de trayectorias en dos tipos de gráficos: gráficos de cronogramas y gráficos de secuencias individuales, respectivamente. En cada uno de estos gráficos, la trayectoria de estrés familiar y de estrés laboral se despliega en diferentes columnas. A la izquierda de los gráficos están representados los nombres y proporciones ponderados de cada tipo de trayectoria, y a la derecha los estatus de estrés familiar y laboral utilizados para reconstruir dichas trayectorias. En el gráfico de cronogramas, el eje X representa la edad de las personas; y el eje Y, la proporción (0 a 1) de personas en diferentes tipos de estatus de estrés en el tiempo. En el gráfico de secuencias individuales, el eje X también indica la edad, pero el eje Y muestra la trayectoria específica de cada individuo clasificado en un tipo de trayectoria (es decir, existen tantas líneas como personas clasificadas en dicho tipo).

**Figura 3:** Gráficos de cronogramas de los cuatro tipos de trayectorias de estrés familiar y laboral.

Nota: En paréntesis se indica la proporción ponderada de cada uno de los tipos de trayectoria de estrés familiar y laboral.

**Figura 4:** Gráficos de secuencias individuales de los cuatro tipos de trayectorias de estrés familiar y laboral.

Nota: En paréntesis se indica la proporción ponderada de cada uno de los tipos de trayectoria de estrés familiar y laboral.

El primer tipo, “Ausencia de estrés familiar, estrés laboral persistente”, incluye al 54,8% de la muestra y corresponde a personas que mayoritariamente describieron no haber sentido estrés por causas familiares, pero sí permanentemente por causas laborales. Este grupo manifiesta haber percibido estrés laboral mayoritariamente a partir de los 20 años de edad de forma constante, lo que disminuye después de los 50 años cuando comienza a aumentar progresivamente la salida del mercado laboral.

El segundo tipo de trayectoria, “Ausencia de estrés familiar, fuera del mercado laboral”, reúne al 18,3% de la muestra y se caracteriza por personas que la mayoría del tiempo en sus vidas se encontraron sin un empleo remunerado, y que no reportaron haber sentido estrés por causas familiares. En este grupo algunas personas reportan haber sufrido estrés laboral en etapas tempranas de la adultez (esto es, de 18 a 30 años de edad), pero luego salieron del mercado laboral.

El tercer tipo de trayectoria, “Estrés familiar persistente, ausencia de estrés laboral”, comprende el 6,9% de la muestra y está compuesto por individuos que mayoritariamente casi no reportaron estrés por causas laborales, pero sí por causas familiares. En este tipo, se clasifican incluso algunas personas que indicaron haber sentido estrés familiar desde la primera infancia, aumentando notoriamente después de los 18 años y manteniéndose constante hasta después de los 60 años.

Por último, el cuarto tipo de trayectoria “Ausencia de estrés familiar y laboral” incluye al 20% de la muestra y se compone de personas que mayoritariamente describieron no haber sentido estrés ni por causas familiares ni laborales en sus vidas.

### Trayectorias de Estrés Familiar y Laboral y ACV

La Tabla 2 muestra el resultado de tres modelos de regresión logística: el primero incluye solo los tipos de trayectorias de estrés, el segundo agrega las variables sociodemográficas, mientras que el tercero considera también los factores de riesgo tradicionales de ACV. Los resultados son reportados en *odds ratios* (OR) con sus respectivos intervalos de confianza y niveles de significancia estadística.

**Tabla 2:** Regresión logística sobre accidente cerebrovascular.

		*Odds ratios*	95% intervalos de confianza	*Odds ratios*	95% intervalos de confianza	*Odds ratios*	95% intervalos de confianza
Tipo de trayectorias de estrés laboral y familiar							
	Ausencia de estrés familiar y laboral						
	Ausencia de estrés familiar, estrés laboral persistente	2,42	0,77—7,56	3,16^d^	0,89–11,17	3,38^d^	1,00–11,50
	Ausencia de estrés familiar, fuera de mercado laboral	9,18^b^	2,16—39,00	8,54^b^	1,80–40,50	8,28^b^	1,86–36,93
	Estrés familiar persistente, ausencia de estrés laboral	12,64^a^	3,53—45,23	12,90^a^	2,96–56,14	11,87^b^	2,80–50,38
Variables sociodemográficas							
Edad				1,09	0,87–1,36	1,11	0,90–1,37
Género							
	Hombre	–		–		–	
	Mujer			0,94	0,44–2,01	0,80	0,37–1,83
Nivel educacional							
	Secundaria o terciaria	–		–		–	
	Primaria			1,95^d^	0,93–4,11	1,50	0,79–2,84
Cobertura de salud							
	Pública	–		–		–	
	Privada			0,00^a^	0,00–0,00	0,00^a^	0,00–0,00
Factores de riesgo tradicionales de ACV							
Índice de masa corporal							
	No obeso	–		–		–	
	Obeso	–		–		1,72	0,74–4,00
Actividad física							
	Una vez al mes o menos	–		–		–	
	Una o más veces por semana		–		–	0,90	0,25–3,19
Consumo de lácteos							
	Menos de una vez por semana	–		–		–	
	Una o más veces por semana					0,51	0,17–1,54
Consumo de legumbres, alubias o huevos							
	Menos de una vez por semana	–		–		–	
	Una o más veces por semana		–		–	0,28^c^	0,09–0,86
Consumo de carne, pescado o ave							
	Menos de una vez por semana	–		–		–	
	Una o más veces por semana	–		–		0,76	0,09–6,40
Hábito tabáquico							
	Ausencia	–		–		–	
	Presencia	–		–		1,21	0,55–2,67
Hipertensión							
	Ausencia	–		–		–	
	Presencia	–		–		–	0,68–4,46
Intercepto		0,02		0,00		0,00	

a p < 0.001.

b p < 0.01.

c p < 0.05.

d p < 0.1. Nota: Efectos estadísticamente significativos marcados en gris.

Como se observa en el modelo que considera los ajustes de todas las variables de control, la posibilidad de sufrir ACV es significativamente mayor en todos los tipos de trayectorias de estrés, en comparación a aquellos sujetos agrupados en la trayectoria “Ausencia de estrés familiar y laboral”. Específicamente, en aquellos sujetos que componen la trayectoria “Ausencia de estrés familiar, estrés laboral persistente”, la probabilidad de sufrir ACV es más de dos veces mayor (OR = 3,38; p < 0,1). Mientras que para aquellos clasificados en la trayectoria “Ausencia de estrés familiar, fuera del mercado laboral” la probabilidad es más de siete veces mayor (OR = 8,28; p < 0,01), y para aquellos que siguieron la trayectoria “Estrés familiar persistente, ausencia de estrés laboral” la probabilidad es más de diez veces mayor (OR = 11,87; p < 0,01). En relación a los factores de control, se observa que el hecho de tener cobertura privada de salud, así como el consumo de legumbres, alubias o huevos más de una vez a la semana, reduce el riesgo de ACV.

Adicionalmente, basados en evidencia previa sobre diferencias entre hombres y mujeres en enfermedades cerebrovasculares^27^, condujimos un análisis de sensibilidad incluyendo efectos de interacción entre los tipos de trayectorias y género sobre el riesgo de ACV (ver Tabla 3). Como se puede observar, el modelo que considera todos los controles sugiere que solo en la relación entre la trayectoria “Estrés familiar persistente, ausencia de estrés laboral” y ACV los hombres muestran un efecto significativamente superior al de las mujeres (OR = 8,69; p < 0,5).

**Tabla 3:** Regresión logística sobre accidente cerebrovascular (efectos de interacción entre tipos de trayectorias y género).

		*Odds ratios*	95% intervalos de confianza	*Odds ratios*	95% intervalos de confianza	*Odds ratios*	95% intervalos de confianza
Tipo de trayectorias de estrés laboral y familiar							
	Ausencia de estrés familiar y laboral	–		–		–	
	Ausencia de estrés familiar, estrés laboral persistente	1,31	0,23–7,35	1,94	0,28–13,18	2,27	0,33–15,58
	Ausencia de estrés familiar, fuera de mercado laboral	7,50	0,59–95,57	7,71	0,47–125,29	8,98	0,59–95,57
	Estrés familiar persistente, ausencia de estrés laboral	8,32^b^	1,52–45,64	9,15^b^	1,37–61,18	8,69^b^	1,22–61,83
Tipo de trayectorias de estrés laboral y familiar * Género							
	Ausencia de estrés familiar, estrés laboral persistente * Mujer	3,54	0,30–42,16	2,72	0,20–36,25	2,28	0,17–28,86
	Ausencia de estrés familiar, fuera de mercado laboral * Mujer	2,27	0,09–46,06	1,64	0,06–44,34	1,27	0,04–36,28
	Estrés familiar persistente, ausencia de estrés laboral * Mujer	2,06	0,15–33,96	2,00	0,14–28,25	1,92	0,12–29,51
Variables sociodemográficas							
Edad				1,09	0,88–1,36	1,12	0,92–1,37
Nivel educacional							
	Secundaria o terciaria	–		–		–	
	Primaria			1,87	0,85–4,01	1,44	0,74–2,80
Cobertura de salud							
	Pública	–		–		–	
	Privada			0,00^a^	0,00–0,00	0,00^a^	0,00–0,00
Factores de riesgo tradicionales de ACV							
Índice de masa corporal							
	No obeso	–		–		–	
	Obeso	–		–		1,70	0,75–3,88
Actividad física							
	Una vez al mes o menos	–		–		–	
	Una o más veces por semana	–		–		0,91	0,26–3,25
Consumo de lácteos							
	Menos de una vez por semana	–		–		–	
	Una o más veces por semana	–		–		0,51	0,17–1,51
Consumo de legumbres, alubias o huevos							
	Menos de una vez por semana	–		–		–	
	Una o más veces por semana	–		–		0,29^b^	0,09–0,88
Consumo de carne, pescado o ave							
	Menos de una vez por semana	–		–		–	
	Una o más veces por semana	–		–		0,76	0,09–6,52
Hábito tabáquico							
	Ausencia	–		–		–	
	Presencia	–		–		1,25	0,58–2,69
Hipertensión							
	Ausencia	–		–		–	
	Presencia	–		–		1,73	0,68–4,40
Intercepto		0,02		0,00		0,00	

a p < 0.001.

b p < 0.05. Nota: Efectos estadísticamente significativos marcados en gris.

## DISCUSIÓN

Utilizando una encuesta retrospectiva, representativa de personas de 65 a 75 años de edad de la ciudad de Santiago, Chile, y basados en un enfoque de curso de vida, este estudio analizó la asociación entre distintos tipos de trayectorias simultáneas de estrés familiar y laboral y la presencia de ACV en la vejez. Los resultados de este estudio indican que una proporción importante de personas mayores en Chile (61,7%) siguieron trayectorias marcadas por la presencia permanente de estrés familiar y/o laboral. Asimismo, una proporción no menor (18,3%) tuvo una trayectoria caracterizada por la ausencia prolongada del mercado laboral.

En términos de la asociación con ACV, observamos que trayectorias caracterizadas tanto por la exposición permanente a situaciones familiares y/o laborales estresantes a lo largo de la vida, se asocian significativamente con la presencia de esta enfermedad cardiovascular. Estos hallazgos son coherentes con estudios transversales o cros-seccionales previos. Por ejemplo, en un metaanálisis de 14 estudios que involucró 10.130 pacientes con diagnóstico de ACV, se estimó un aumento del riesgo del 33% en esta patología entre aquellos que reportaron sentir estrés por motivos familiares, laborales o financieros^28^.

Nuestros resultados muestran en particular que el tipo de trayectoria caracterizado por la presencia persistente de estrés vinculado a causas familiares, pero con ausencia de estrés laboral, se asocia más a la presencia de ACV (indicando un riesgo más de 10 veces superior al tipo de trayectoria “Ausencia de estrés familiar y laboral”). Este resultado se ajusta adecuadamente a estudios previos que han observado un efecto relevante sobre la presencia de ACV en aquellas personas que enfrentan relaciones maritales e interpersonales estresantes^29^. Además, nuestros análisis de sensibilidad (Tabla 3) nos muestran que este tipo de trayectoria es significativamente más riesgosa en hombres que en mujeres. Al respecto, algunas investigaciones han apuntado un aumento del riesgo de sufrir ACV en grupos que habían experimentado estrés asociado al divorcio o fallecimiento del cónyuge, particularmente entre hombres^14^.

Por otro lado, nuestro estudio evidencia una fuerte asociación con ACV entre personas que reportan no haber sufrido estrés por causas familiares, pero estaban fuera del mercado laboral durante períodos prolongados. Estos resultados también coinciden con estudios transversales previos, que muestran que aquellas personas que dejan de trabajar remuneradamente tienen más del doble de riesgo de padecer ACV (así como otras enfermedades cardiovasculares como el infarto agudo al miocardio), especialmente si la pérdida del empleo se produce de forma involuntaria^27,30,31^. Además, otros estudios en Latinoamérica indican una mayor mortalidad asociada a ACV en sujetos inactivos^32^. Una posible explicación de esta asociación corresponde a que, sobre todo en países como Chile, tener un empleo de manera continua implica obtener a un ingreso financiero que permite satisfacer necesidades individuales tales como el acceso a mejores servicios preventivos de salud y a tratamientos farmacológicos^33–35^.

### Limitaciones

Este estudio tiene ciertas limitaciones que deben ser consideradas. En primer lugar, debido al ineludible sesgo de memoria, los encuestados podrían haber reportado de forma inexacta experiencias tempranas de su vida. Para mitigar este sesgo, el calendario de curso de vida empleado en este estudio incorporó innovaciones metodológicas orientadas a fortalecer la memoria autobiográfica, tales como la descomposición de los dominios de vida en diferentes subcategorías, el uso de ayudas visuales y la orientación de un encuestador debidamente capacitado en el proceso de rellenado del cuestionario. En relación a la muestra, es relevante considerar que esta es representativa de la capital, pero no de todo Chile. Esto es importante, por cuanto el centralismo del país genera que el acceso a los servicios de salud sea mayor y de mejor calidad en la capital que en el resto de las regiones. Una última limitación relevante refiere a que el reporte de estrés por parte de los encuestados depende estrechamente de su percepción del concepto “estrés”. Sabemos, sin embargo, que no existe una definición única de este término, pues abarca aspectos psicológicos, emocionales, motivacionales y cognitivos. Asimismo, al momento de identificar estrés laboral, los participantes pueden haber considerado aspectos diferentes que cuando reportaron estrés familiar.

## IMPLICANCIAS Y CONCLUSIÓN

Este estudio tiene implicancias relevantes para el campo de enfermedades cardiovasculares. Los ACV son patologías de alta prevalencia, con consecuencias catastróficas en la salud y altamente asociadas a factores de riesgo modificables, entre ellos el estrés. Para disminuir la mortalidad y las secuelas que estas patologías tienen en la población, es fundamental considerar dichos factores de riesgo y fortalecer los sistemas sanitarios con un enfoque promocional, preventivo, con eje en la disminución de las inequidades en salud, y no solo en la optimización de tecnologías médicas de tratamiento y rehabilitación.

Como sugiere este estudio, el estrés es un factor de riesgo que experimenta de manera prolongada una alta proporción de personas en distintas etapas y dominios de la vida. En consecuencia, es necesario adoptar un enfoque promocional y preventivo que enfatice los efectos adversos de experimentar cotidiana y acumulativamente situaciones estresantes. La formulación de políticas públicas orientadas a reducir el estrés podría contribuir a mitigar el riesgo de ACV en poblaciones tradicionalmente susceptibles.
